# Towards audio-based identification of Ethio-Semitic languages using recurrent neural network

**DOI:** 10.1038/s41598-023-46646-3

**Published:** 2023-11-07

**Authors:** Amlakie Aschale Alemu, Malefia Demilie Melese, Ayodeji Olalekan Salau

**Affiliations:** 1 Department of Electrical and Computer Engineering, Gafat Institute of Technology, Debre Tabor University, Debre Tabor, Ethiopia; 2 Department of Information Technology, Gafat Institute of Technology, Debre Tabor University, Debre Tabor, Ethiopia; 3 Department of Electrical/Electronics and Computer Engineering, Afe Babalola University, Ado-Ekiti, Nigeria; 4 Saveetha School of Engineering, Saveetha Institute of Medical and Technical Sciences, Chennai, Tamil Nadu India

**Keywords:** Medical research, Materials science

## Abstract

In recent times, there is an increasing interest in employing technology to process natural language with the aim of providing information that can benefit society. Language identification refers to the process of detecting which speech a speaker appears to be using. This paper presents an audio-based Ethio-semitic language identification system using Recurrent Neural Network. Identifying the features that can accurately differentiate between various languages is a difficult task because of the very high similarity between characters of each language. Recurrent Neural Network (RNN) was used in this paper in relation to the Mel-frequency cepstral coefficients (MFCCs) features to bring out the key features which helps provide good results. The primary goal of this research is to find the best model for the identification of Ethio-semitic languages such as Amharic, Geez, Guragigna, and Tigrigna. The models were tested using an 8-h collection of audio recording. Experiments were carried out using our unique dataset with an extended version of RNN, Long Short Term Memory (LSTM) and Bidirectional Long Short Term Memory (BLSTM), for 5 and 10 s, respectively. According to the results, Bidirectional Long Short Term Memory (BLSTM) with a 5 s delay outperformed Long Short Term Memory (LSTM). The BLSTM model achieved average results of 98.1, 92.9, and 89.9% for training, validation, and testing accuracy, respectively. As a result, we can infer that the best performing method for the selected Ethio-Semitic language dataset was the BLSTM algorithm with MFCCs feature running for 5 s.

## Introduction

In reality, people's use of natural language as a way of exchanging information has progressed to the point that it has become essential for technological advancement^1^. The interest in using technology to decipher natural language in order to make crucial information available to the public has grown tremendously in recent years. In response to this need, natural language processing (NLP) has been a primary focus of natural language computations^2^. The waveform or speech signal contains the overall message as well as the speaker's features and the language of interaction. The structure of sound units indicates the language. The goal of automatic language identification (LID) is to identify a language from a spoken speech. This domain of NLP performs significant research in the area of language identification^3,4^.

Almost all Ethiopian languages lack resources, placing them among the language groups that do not benefit from the most recent advances in spoken language technology^5^. The current effort focuses on Ethiopian Semitic (Ethio-Semitic) languages. According to recent linguistics research, Semitic languages are classified as the parent category of the Afro-Asiatic category. This categorization is supported by a large number of famous linguists^1–5^. There are up to 200 dialects spoken in Ethiopia, with around 86 languages divided into the Semitic, Cushitic, Omotic, and Nilo-Saharan groups^6^. The Ethio-Semitic language family includes Amharic, Tigrigna, Geez, Harari, Gafat, Soddo, and Gurage^7^.

However, for our research, we employed Amharic, Geez, Gurage, and Tigrigna because they all have a sizable number of native speakers. These languages are used for a variety of reasons in Ethiopia. Amharic is the working language of both the Amhara regional state and the Federal Government. Tigrigna is the official language of the 9 million people in the Tigray regional state and its also Eritrea's official language. Ge'ez is Ethiopia's ancient language, and it is still used for religious rites by Jews, Beta Israel, and Ethiopian Christians. The Ethiopic or Ge'ez script is used to write the Gurage languages^5,6^. In this paper, we have employed a unique deep learning method for Ethiopian LID. Recurrent neural networks (RNN) are excellent at handling sequential data, such as that found in audio and music^7^. RNN incorporation in this work allows the preceding layer's output data to be considered. We used the most crucial and strong audio to identify sound. Mel-frequency cepstral coefficients (MFCC) is a method for extracting information which we used to aid the proposed system to reliably identify the selected languages^8^.

## Background of deep learning approaches

### Overview of recurrent neural network

Recurrent neural network (RNN) is effective in modeling temporal relationships in the audio signals in order to build long-range feature representations throughout the input sequence for language detection. RNN was first proposed by Elman in 1990^9^. RNN is a model in which the output is a function of not only the current input but also the previous output, which is encoded into a hidden state. RNN has a memory of the preceding timestep, which it uses to encode the information in the sequence^9–11^. As a result, by taking into consideration the entire temporal sequence, RNN can model multivariate time-series data and can provide a class prediction.

### Long short-term memory

Memory blocks are peculiar items found in the recurrent hidden layer of long short-term memory (LSTM). The memory blocks feature exceptional multiplicative units known as gates that control the flow of information as well as memory cells with self-connections that record the network's temporal state. The input gate regulates the flow of input activations into the memory cell, while the output gate controls how cell activations exit the cell and enter the network. The forget gate scales the internal state of the cell before adding it as input via the cell's self-recurrent link, adaptively forgetting or resetting the cell's memory^11,12^.

### Bidirectional long short term memory

Bidirectional long short-term memory (BLSTM) is a kind of bidirectional recurrent neural network (BRNN). It was originally proposed in^9^ and comprises of a combination of two hidden layers that expose separate directions to the same output. The output layer can gather information from both previous (backwards) and forward (future) states at the same time. Each LSTM is made up of increasingly complex and often linked subnets called "memory cells." These cells (gates) enable information to have long-distance dependencies. As a result, this adds fresh values to the activation as it progresses through the layers, avoiding the vanishing gradient problem that makes LSTM comparable to residual neural networks, or ResNets^13,14^.

Numerous research on language identification (LID) have been undertaken in the past which used conventional machine learning and deep learning approaches. The following are the study's contributions:

- There is no publicly available dataset for the LID of Ethio-Semitic languages. In this study, we created the first audio dataset to address this issue. As a result, this dataset may be utilized for future research and to compare similar works.
- In addition, we presented an efficient RNN model with appropriate hyper-parameters.
- Finally, we achieved state-of-the-art LID accuracy for Ethio-Semitic languages using the proposed method.

The rest of the paper is structured as follows: Section "Background of the deep learning approaches" presents a detailed description of the related work, including the gaps and limitations used in this study. Section "Related work" provides a detailed description of the proposed methodology and the dataset analyzed in this study. The experimental findings and analysis are provided in Section "Methodology". Finally, we present the concluding remarks in Section "Results and Discussion".

## Related work

In an attempt to identify the gap in knowledge in this study area, we examined past research which used the conventional machine learning and deep learning approaches. This enabled us identify the gap in knowledge, existing datasets and sizes, methodologies, and models employed by various authors. Wondimu and Tekeba^3^ used Gaussian mixture model (GMM) in a LID system for four Ethiopian languages (Amharic, Oromiffa, Guragegna, and Tigrigna). In the study, the MFCC feature extraction approach was employed, while GMM was used for classification. There is no fixed duration or segment size for audio splitting in the study. For the four languages, the average accuracy of the utterance dependent LID test was 93%; the average accuracy of the utterance independent test was around 70%; and the average accuracy of the speaker independent test, which was only evaluated on the utterance dependent scenario, was around 91%.

Athiyaa et al*.*^4^ suggested using the Gaussian Mixture Model (GMM) and Mel-Frequency Cepstral Coefficients (MFCCs) aspects of the speech waveform to discriminate between two independent audio signals in Tamil and Telugu, two South Indian languages. The suggested approach uses MFCC features taken from speech waveforms to train the Gaussian Mixture Model (GMM). With an additional blend feature, the proposed spoken language recognition method improves the accuracy for both languages.

Gonzalez-dominguez et al*.*^12^ research study showed how LSTM RNNs effectively exploits temporal correlations in audio data by learning appropriate features for language recognition. The suggested method is compared to the i-vector and feed-forward Deep Neural Network (DNN) baseline methods on the NIST Language Recognition Evaluation 2009 dataset. Despite having less magnitude values of the parameters, their results demonstrate that LSTM RNNs outperformed the DNN system. Additionally, the fusion of the various technologies enables significant speed gains of up to 28%.

Bartz et al*.* ^15^ method used a multi-head self-attention layer, an encoder, and the statistics pooling layer. The encoder effectively includes information from the raw waveforms using 1D kernels and an LSTM layer. The multi-head self-attention layer uses the outputs from the LSTM layer to apply self-attention mechanisms with different heads to these characteristics. Through this approach, the model is capable of providing more weight to the features that are more relevant and less weight to the features that are less essential. According to their investigation, they found that their method outperformed the 373 h of audio data for eight distinct Indian languages and that it greatly outperformed the benchmark model in terms of F1-score, achieving a score of 95.90%. Their study shows that anytime raw waveform models were applied, the performance increased by 1.7%.

Deshwal et al*.*^16^ used a hybrid robust feature extraction technique for spoken language identification (LID) systems. In the feature extraction stage, different techniques are applied individually, such as Mel frequency cepstral coefficients (MFCCs), perceptual linear prediction features (PLP), and relative perceptual linear prediction features (RASTA-PLP). The feed forward back-propagation neural network (FFBPNN) was used in the language identification or classification phase. With an overall accuracy of 94.6%, the MFCC-RASTA-PLP hybrid feature extraction approach shows promising results when compared to the other hybrid feature extraction techniques.

Anjana and Poorna^17^ used Linear Discriminant Analysis (LDA) and Support Vector Machine (SVM) as classifiers to extract languages from speech data. The MFCC and Formant features of Speech were used in the study to compare and contrast the two feature types with the two models separately. With just MFCC and formant frequencies, the accuracy of SVM and LDA was 78%, 86%, and 80.02 & 93.06%, respectively. The LDA model, which integrates the two features, performs better, achieving 93.88% accuracy compared to SVM's 84%. While other authors only examined one or two different languages, in this paper, we examined four languages. It is noteworthy to state that there is no existing dataset for Ethio-Semitic languages in the body of existing knowledge online. In addition, authors have used methods which have not been used before such as RNN, Long Short Term Memory (LSTM) and Bidirectional Long Short Term Memory (BLSTM) for the identification of Ethio-Semitic languages.

## Methodology

The methods to achieve successful results and achieve the goals of this research are described in this section. The sections of the study include subsections such as data collection and annotation, audio preprocessing, data splitting, feature extraction, model training, and model evaluation. Furthermore, an endeavor is made to elaborate on the study's unique feature, the identification of particular Ethio-Semitic languages.

### Data collection

There is no publicly available dataset of audio data from Ethio-Semitic languages. Even though there is no prepared data for the selected languages, we created our own dataset by recording directly from native speakers of each language in order to execute the automatic language identification task. With a record time of 12 min per person for each language, we used 10 people for each language. Each corpus is composed of a variety of audio recordings containing different ages, genders, and accents. Each language's recording lasted for about 120 min (2 h), thereby totalling 480 min (8 h). The speech signal has a sampling rate of 44.1 kHz, and each sample is stored as a 16 bit value. This was recorded using a smart phone.

### Proposed system architecture

**Figure 1:**
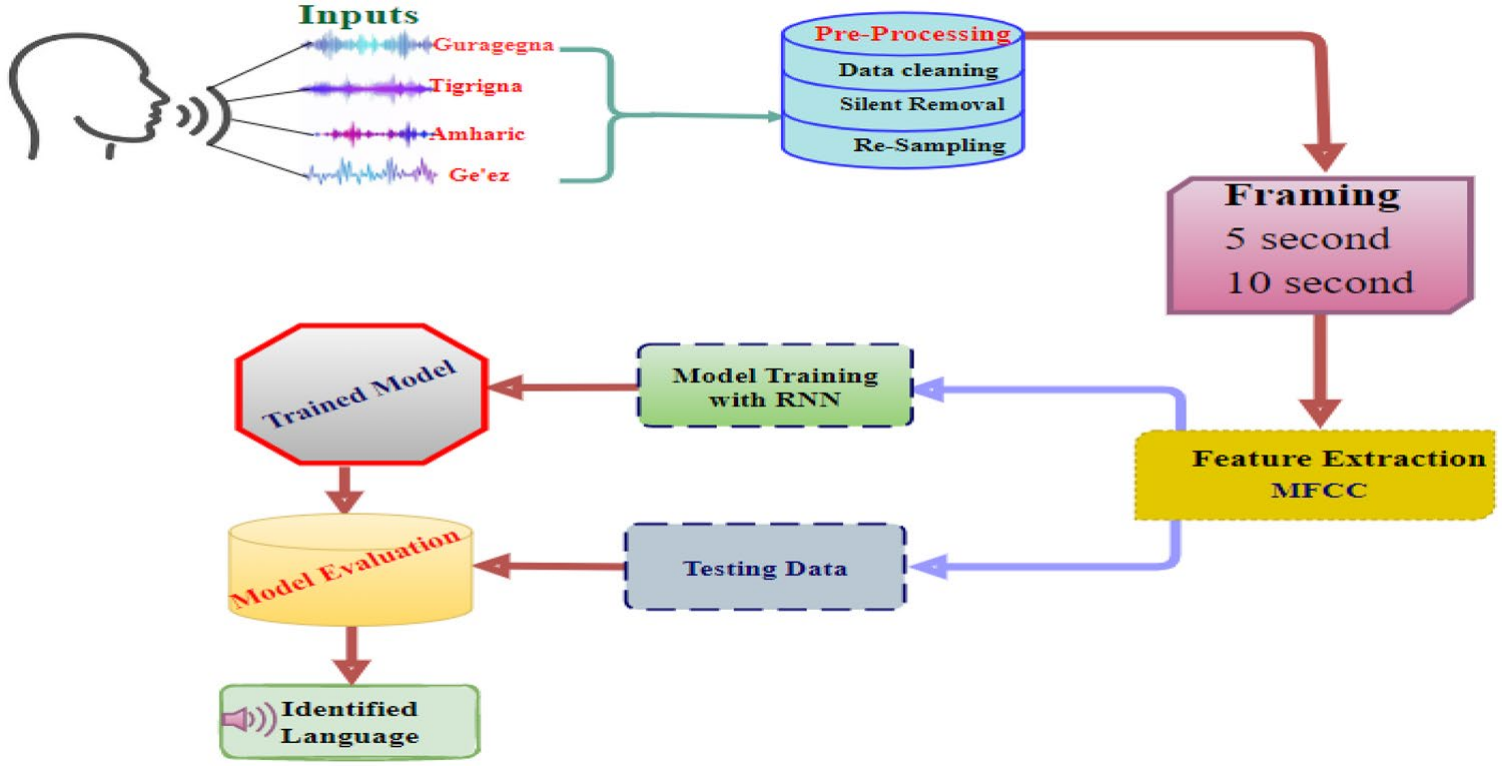
Proposed system architecture.

This section describes the steps undertaken to build the proposed LID model using the proposed architecture in Fig. 1. It primarily focuses on the corpus preparation, overall model system architecture, preprocessing methods, preparation of machine readable datasets (CSV), and model evaluation procedures. Figure 1 depicts the proposed LID model’s architecture where the model consists of a number of steps such as data preprocessing, framing, feature extraction, and model evaluation.

#### Data Pre-processing

After data collection, the first step in the LID system is to pre-process the raw speech data to make it suitable for the study. It is the term used to describe any type of processing performed on raw data to get it ready for the next processing step. It is the primary step that converts the data into a format that can be processed more quickly and efficiently.

##### Data cleaning

Audio data cleaning is mandatory before any processing steps. When we collect the data by recording, most of the time the speakers speak mixed languages like Amharic with English or Ge’ez with Amharic, etc. Therefore we remove those mixed audios by using pydub package to prepare pure data for the proposed system. The audio files were saved in the form of WAV files.

##### Silent Removal

We used librosa trim function in order to remove the silence from the actual speech wav files and the normal sound is greater than 10 dB^18^. Decibel (dB) measures sound intensity (amplitude). We utilized a 10 dB threshold to eliminate the silence from speech. As a result, any speech that falls below this threshold is erased from the whole sound file^18^. We used 10 dB as we reviewed numerous literature which used the same level of sound. This is a standard value as the sound must be greater than the noise as given by Signal-to-Noise ratio (-10 dB ≤ SNR < + 10 dB). Also any speech which is greater or equal to 10 dB provides an adequate quality for speech communication.

##### Resampling

Even though our data was initially sampled at 44,100 Hz and had a large data size, in a comparison of 44,100 Hz and 22,050 Hz, the former has much greater data size. Because of the large amount of the data, the dataset was initially down-sampled to 22,050 Hz from 44,100 Hz. All audio files were converted from stereo (two channel) to single-channel (mono) since mono channel is superior for language classification and a sampling rate of 22.05 kHz is sufficient for basic information^19,20^. Each audio recording in stereo channels contains two channels, and is less essential for language classification than mono channels^21,22^. Librosa's default settings include a sampling rate of 22050 Hz, a bit depth of 16 bits, and 1 channel (mono).

#### Framing

Framing is the technique of splitting a continuous stream of speech samples into units of fixed length that enables it to process the signal block-by-block. Speech signals are slowly time-varying or non-stationary. Since sound waves are non-stationary signals, their acquired data are constantly shifting over time. Consequently, it is not possible to extract the speech features at once^23^. The speech signal is broken down into frames, which are small period subdivisions in second or millisecond^8^. Consecutive frames usually overlap by 30 to 50% in order to avoid windowing from obliterating any essential voice signal information^24^. Different segment lengths can be applied, namely: 30 s, 20 s, 10 s, 5 s and 3 s^25^. We selected intermediate lengths which are, 5 s and 10 s to segment or for framing using audio segment library for each language sound.

#### Feature extraction

The method of converting a raw speech signal into a collection of acoustic feature vectors that carry speaker-specific information is referred to as feature extraction^26^. By using Librosa package, we extract useful components from audio data^27,28^. There are different feature extraction techniques like Chorma Stft, Mel-frequency cepstral coefficients** (**MFCC), mel-spectrogram, Spectral Contrast, and Tonnetz etc.^27,28^. There are numerous features of an audio speech segment that can vary from language to language. These can be included into various LID system designs, each with a unique level of complexity and outcome^29^. Several feature extraction techniques exist for language classification^30^. The proposed system employs an acoustic-phonetics feature type with feature extraction technique namely MFCC have been used for feature extraction which is mainly used for spoken language identification.

#### Mel-frequency cepstral coefficients feature

**Figure 2:**
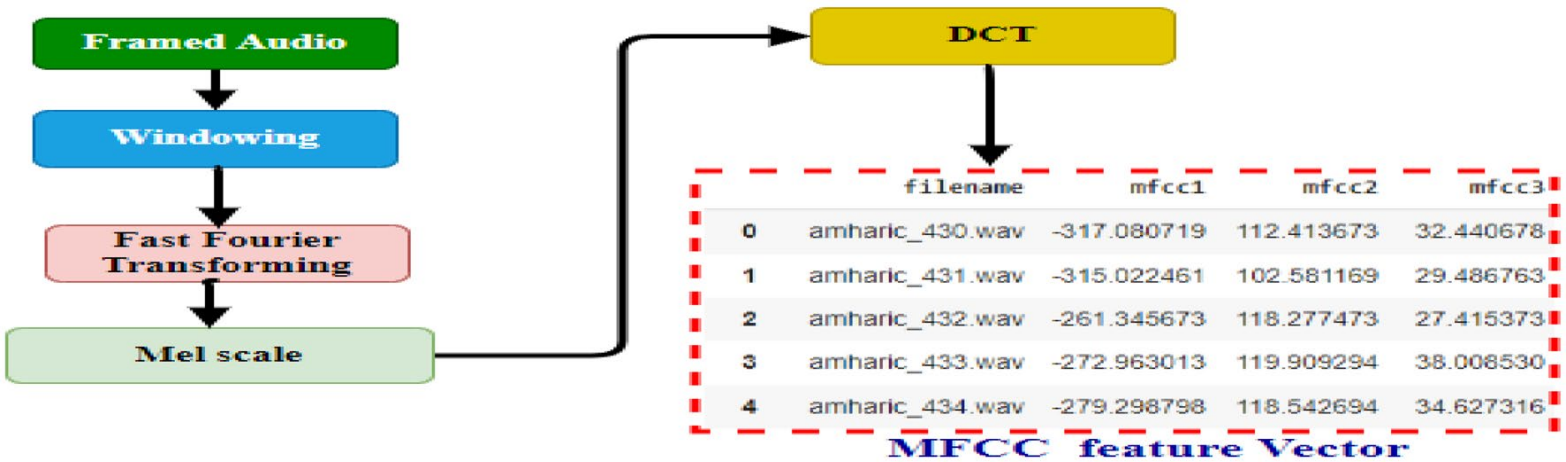
MFCC block diagram.

The most common feature extraction technique to represent speech signal data and to express acoustic signals as cepstral coefficients for various applications is Mel-frequency cepstral coefficients** (**MFCCs)^16^. MFCC is a well-known feature used to describe speech signals. Since the technique for computing MFCC is based on short-term analysis, a MFCC vector is generated from each frame. These are based on speech processing performed by the human ear and the speech signal's cepstrum^30,31^. Based on the study in^21^, we took the number of MFCC coefficients as 20, since the most important information were found from 13 to 20 coefficients^8,21,32^. The process to extract MFCC features is shown in Fig. 2.

### A. Windowing

Windowing is the first step to extract MFCC features from framed signals and the effect of windowing is used to convert an infinite-duration signal into a finite-duration signal^33^. The signal should be attenuated to zero or very similar to zero in order to reduce the discontinuity of the voice signal at the start and end of each frame. Therefore, windowing each frame of the signal to increase the correlation of the Mel Frequency Cepstrum Coefficients (MFCC) can be used to minimize the difference^24^.

### B. Fast fourier transform

Fast Fourier Transform (FFT) is used to convert the data from the frequency domain to the spatial domain. The samples in each frame are converted into frequency domain. A fast algorithm for performing the discrete Fourier transform is the Fourier transformation (DFT) ^33,34^.

### C. Mel-scale

In this stage, the projected spectra from the previous phase are mapped on the Mel scale to produce an approximation of the energy present at each point using a triangle overlapping window, also known as a triangular filter bank^8,21,22,33,34^.

### D. Discrete cosine transform

**Figure 3:**
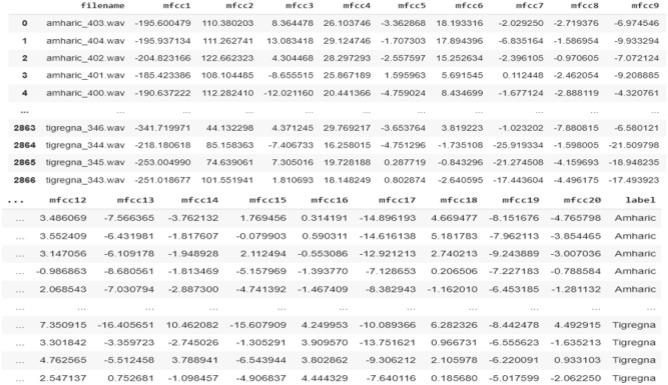
Sample of MFCC vector feature.

This is the final stage in the process of converting a given sequence of finite duration data into a discrete vector by calculating coefficients from the provided log Mel spectrum. Discrete Cosine Transform (DCT) is preferred for the coefficient calculation since its outputs can have significant energy contents. Finally, the result of applying DCT is referred to as the MFCC vector ^8,23,30^. The discrete cosine transform provides cepstral coefficients and is based on the linear transformation principle (MFCCs)^16^. The sample MFCC vector feature is shown in Fig. 3.

#### Model training

Long-Short Term Memory (LSTM) and Bidirectional Long-Short Term Memory (BLSTM) models were used to develop the proposed system. The MFCC features are fed into the proposed recurrent neural networks as input. The acquired audio dataset was used to train the models.

##### Proposed LSTM model

**Figure 4:**
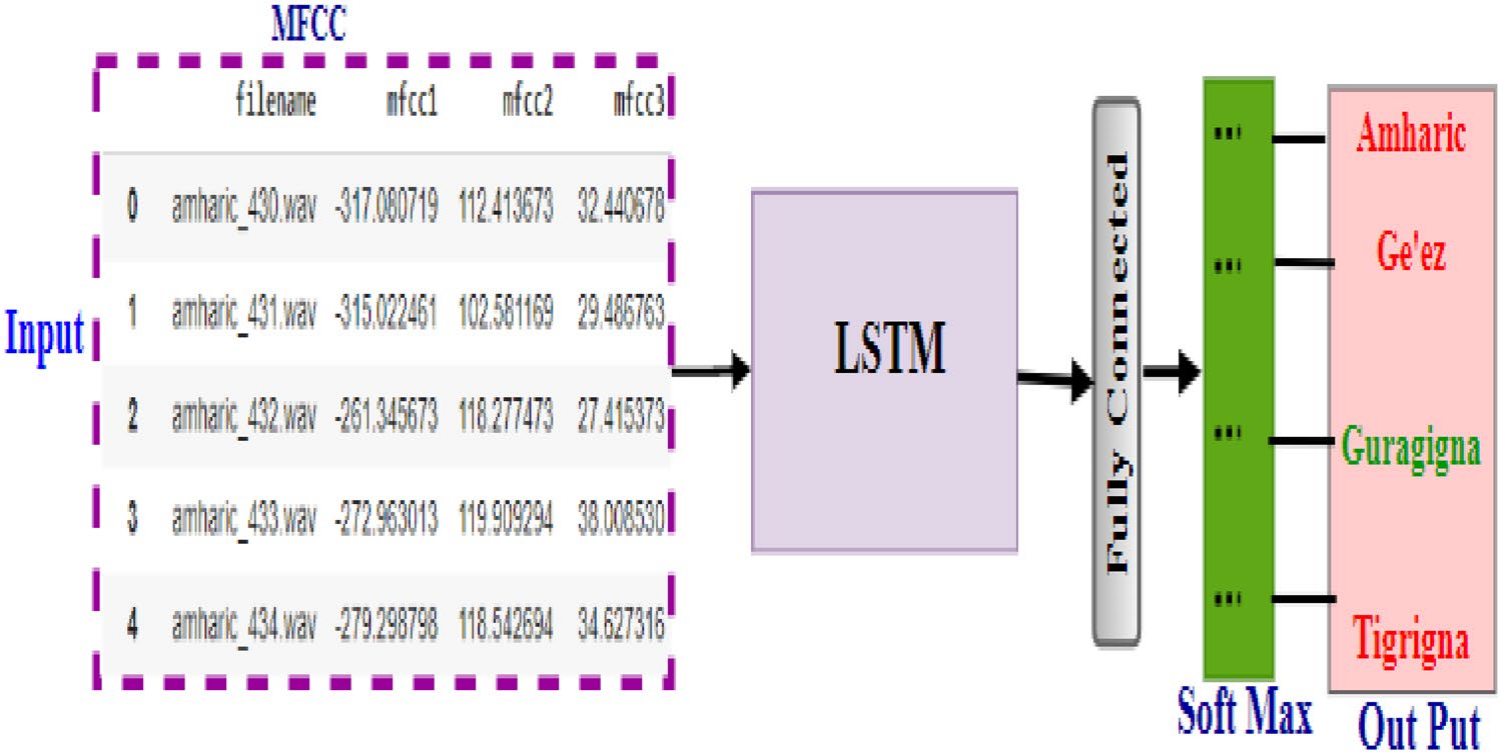
Proposed LSTM model.

The LSTM model is better for sequential data, so therefore, we used the sequential feature value which is MFCC. We fed the MFCC feature vector form directly to the LSTM model^36^. So, using the specified feature extraction technique, we ran one experiment using this model. Figure 4 depicts the proposed LSTM model.

##### Proposed BLSTM model

**Figure 5:**
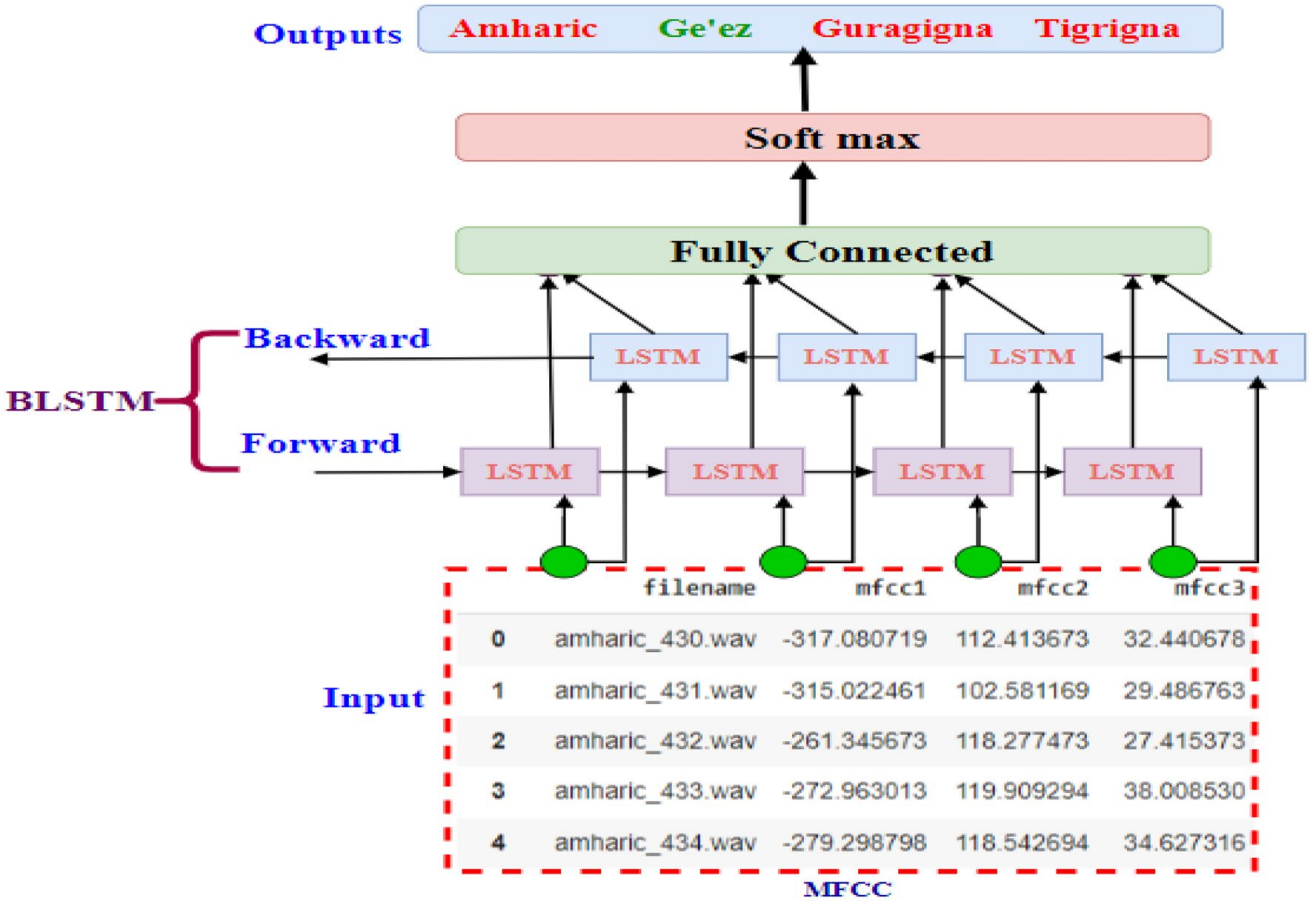
Proposed BLSTM model.

Experimentally, the BLSTM model, like the LSTM model, is better for sequential data. We used the sequential feature, which is similar to the MFCC feature. We fed the MFCC feature vectors directly into the BLSTM model. So we run one experiment with the MFCC feature in this model and used 64 and 128 filters, respectively. Figure 5 depicts the proposed BLSTM model.

#### Model evaluation metrics

There are two types of classification problems depending on the number of classes: binary classification, where there are only two classes, and multi-class classification, where there are more than two classes^37,38^. The confusion matrix is the visual representation of actual and predicted class values. The actual class indicates the real classification of each language and the predicted class also the prediction values of our trained models. Confusion matrix evaluates the accuracy of an algorithm to arrange samples in the appropriate classes^2^. This study used multi-class classification to evaluate the proposed models with four languages and we used evaluation metrics to evaluate the performance which include accuracy, precision, recall, and F1 score given by Eqs. (1)–(4).

1 $$\mathrm{Accuracy}=\frac{\mathrm{TP}+\mathrm{TN}}{\mathrm{TP}+\mathrm{FN}+\mathrm{FP}+\mathrm{TN}}$$

2 $$\mathrm{Precision}=\frac{\mathrm{TP}}{\mathrm{TP}+\mathrm{FP}}$$

3 $$\mathrm{Recall}=\frac{\mathrm{TP}}{\mathrm{TP}+\mathrm{FN}}$$

4 $${\mathrm{F}}_{\mathrm{Score}}=2*\left(\frac{\mathrm{Precision}*\mathrm{Recall}}{\mathrm{Precision}+\mathrm{Recall}}\right)$$

where, TP, TN, FP and FN represent true positive, true negative, false positive and false negative respectively.

### Experimental setup

After feature extraction, we split the dataset into training, validation, and testing data to train, validate and evaluate the model. From the Scikit learn library, we used the train test split method to divide the data into 70% train set, 15% test set, and 15% validation set. The models used and experiments performed in this study are presented in this section along with the results used to evaluate how well the system performed in relation to other systems. To accomplish this, we first evaluated the dataset we used to build our model, then explained the results of that implementation, and finally evaluated the results using the four metrics in Eqs. (1)–(4). Bayesian hyper-parameter tuning algorithm was used to set values for hyper-parameters in our neural network (NN) model. This helps us to find a vector of hyper-parameters that works well with the problem domain in order to determine which one would perform effectively. Hyperparameters including batch size, learning rate, and dropout rates were optimized. We used the hyper-parameters presented in Table 1.

**Table 1:** Hyper-parameter selection summary.

Hyper-parameters	Optimal value
Learning rate	0.001
Optimizer	Adam
Drop out	0.2
Batch size	32
Epoch	35

### Human ethics statements

Authors confirm that all experiments were performed in accordance with the Helsinki declaration guidelines and regulations. Authors confirm that informed consent was obtained from all participants. Authors confirm that all experimental protocols were approved by the institutional licensing/research ethics committee of the author’s institution which include members of staff of the College of Informatics, University of Gondar, Ethiopia, namely: Tsehaye Wasihun, Yigezu Agonafir, Solomon Zewdie, Sied Hassen, Ibrahim Gashaw.

## Results and discussion

This section presents the implemented models as well as the experiments, results produced to evaluate the models performance and the comparison of the various deep learning methods. To evaluate our model, we analyzed the dataset using the deep learning approaches that we utilized to create our model. The results of the implementation and the metrics used to evaluate the results produced are presented in this section. When we examined the performance with durations of 5 s and 10 s, we observed that the performance with durations of 5 s outperforms that of 10 s. The results of the experimentations when LSTM with MFCC is used as a feature is presented in Table 2, while that which used BLSTM with MFCC as a feature is presented in Table 3.

**Table 2:** Experimental result using LSTM with MFCC as feature.

Languages	Duration	Precision (%)	Recall (%)	F1-Score (%)
Amharic	5 s	93	92	93
	10 s	94	89	91
Geez	5 s	92	95	94
	10 s	92	91	92
Guragigna	5 s	90	92	91
	10 s	82	86	84
Tigrigna	5 s	88	87	88
	10 s	84	82	83

**Table 3:** Experimental result using BLSTM with MFCC as feature.

Languages	Duration	Precision (%)	Recall (%)	F1-Score (%)
Amharic	5 s	95	94	95
	10 s	90	95	93
Geez	5 s	94	97	95
	10 s	96	94	95
Guragigna	5 s	92	92	92
	10 s	85	80	82
Tigrigna	5 s	93	90	91
	10 s	81	83	82

For 5 s, Guragigna achieved a precision, recall, and f1-score of 90, 92, and 91, respectively, and for 10 s, it achieved a precision, recall, f1-score of 82, 86, and 84. As a result, it can be deduced that the length of 5 s was superior to that of 10 s.

As shown in Table 3, the developed BLSTM model using MFCC as a feature achieved a recall of between 80 and 95% lasting 10 s and between 90 and 97% lasting 5 s. As a consequence, when the results of 5 s and 10 s are compared, the findings presented in Table 3 surpass the later. As a result, it can be concluded that 5 s was preferred over 10 s.

**Figure 6:**
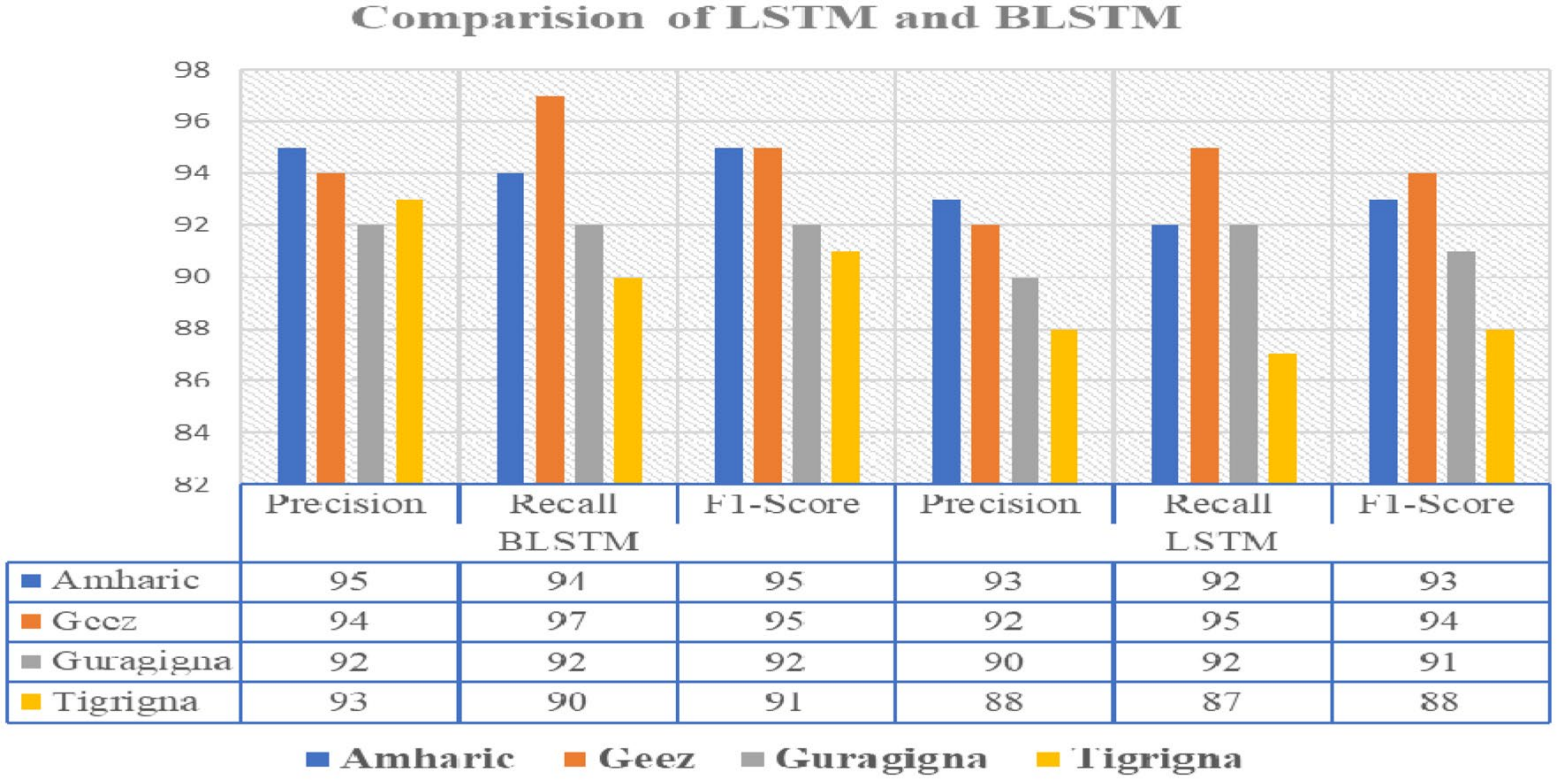
comparison of BLSTM and LSTM using MFCC features.

As illustrated in Fig. 6, BLSTM outperforms LSTM with respect to the LID models performance when employing the MFCC features. As a result, we can infer that the BLSTM is a more crucial algorithm than the LSTM for the building of LID models. With an average of 93.5%, 93.25%, and 93.25% for Precision, Recall, and F1-score, respectively, we achieved better results as compared to existing systems. Generally, for the two models that we proposed, the result of 5 s was greater than 10 s. The reason behind this is that, it is essential to evaluate speech for a short time frame to execute a stable acoustic feature.

### Confusion matrix of the proposed models

**Figure 7:**
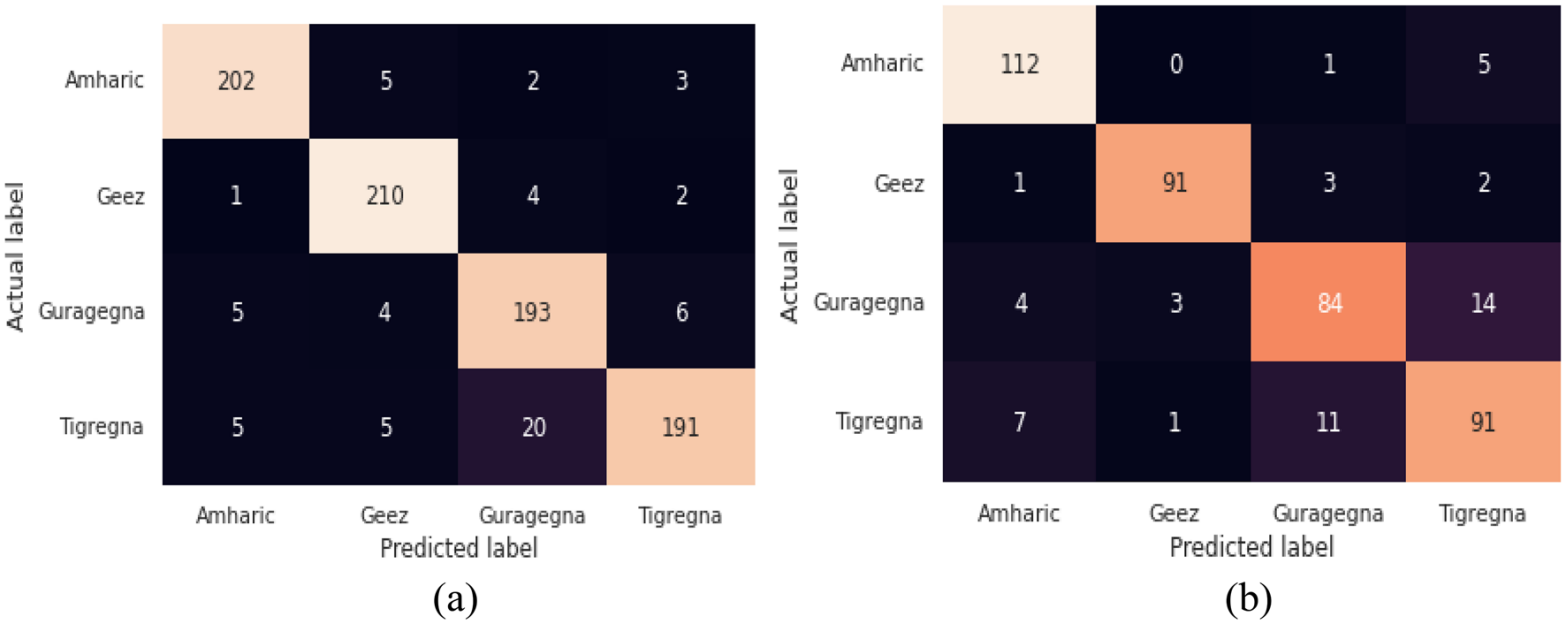
Confusion matrix of BLSTM model (**a**) 5 s (**b**) 10 s.

When the models use the confusion matrix to make predictions for each language, we observed how it becomes confused. We discussed the proposed models' confusion matrix with a specific feature, the MFCC feature. Figure 7 describes the confusion matrix of which the model is examined using the test data set by calculating the number of correctly and incorrectly classified test samples within each class.

 Figure 7(a, b) displays the percentage of sample data that are classified as True Positive (TP), False Positive (FP), True Negative (TN), and False Negative (FN) in each of the four classes. Geez is taken as an illustration for the confusion matrix. It has the true positive value of 210, false-positive value 14, true negative value of 627, and false negative value of 7. Therefore, 210 samples are classified into the Amharic category, 1 sample is misclassified as Amharic, 4 samples are misclassified as Guragigna, 5 samples are misclassified as Tigrigna.

**Figure 8:**
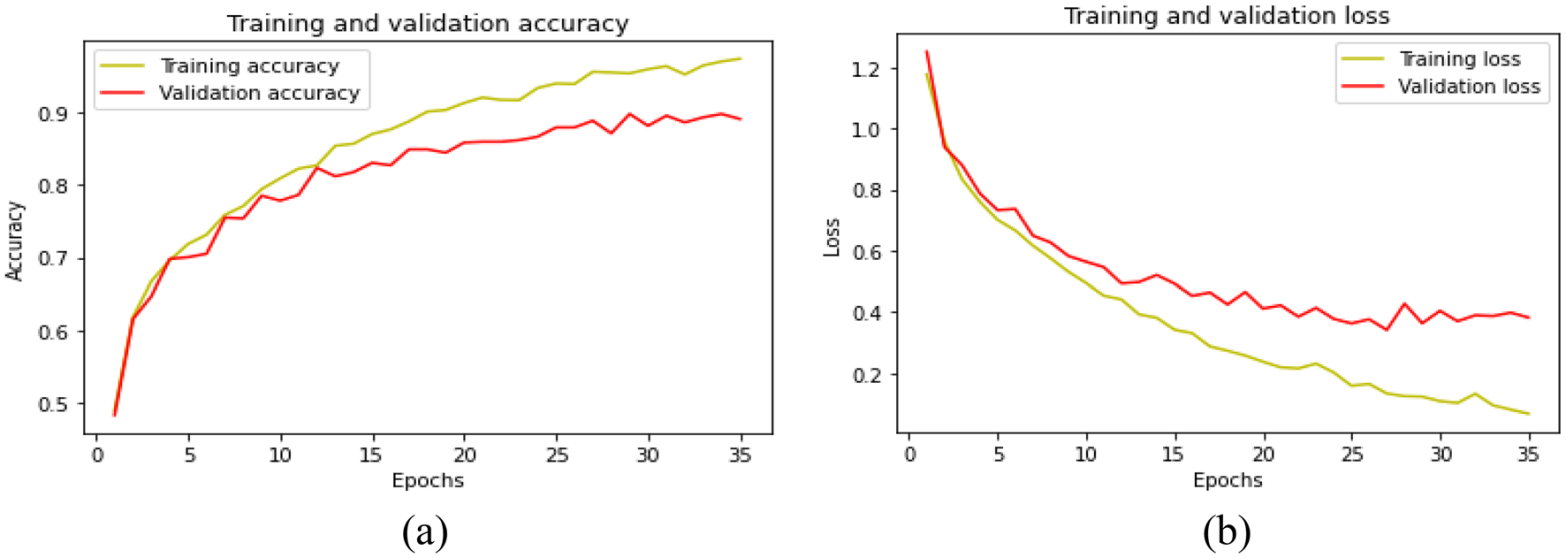
Evaluation metrics of LSTM model with duration of 5 s. (**a**) accuracy (**b**) loss.

Furthermore, Fig. 8 clearly illustrates the accuracy and cross-entropy (loss) performance evaluation of the LSTM model during the training and validation phases. At epoch 35, the accuracy for training and validation is 92.50% and 89.16%, respectively. Similarly, the LSTM architecture's training and validation losses are 0.0673 and 0.3820, respectively.

**Figure 9:**
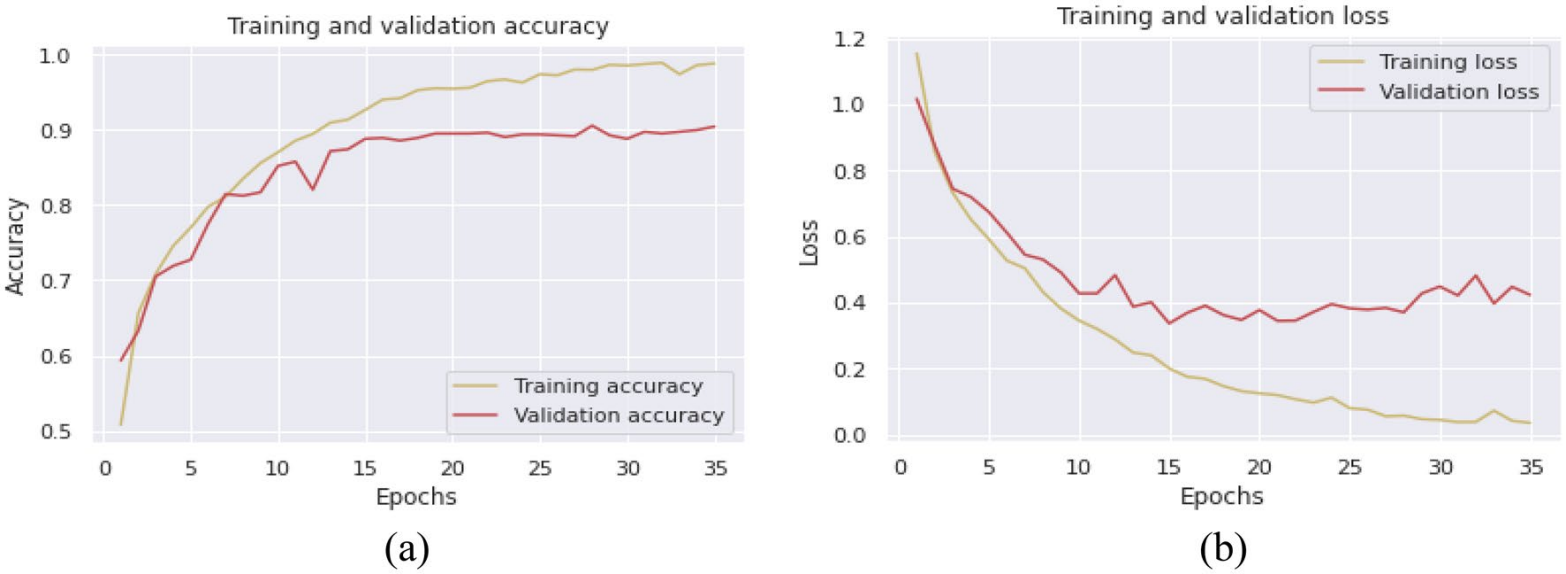
Evaluation metrics of BLSTM model with duration of 5 s (**a**) accuracy (**b**) loss.

Figure 9 presents a visual display of the accuracy and cross-entropy (loss) performance evaluation of the BLSTM classifier during the training and validation periods. At epoch 35, the observed training and validation accuracy are 94.5% and 90.33%, respectively. Also, the BLSTM architecture's training and validation losses are 0.0187 and 0.4231, respectively. The BLSTM architecture surpassed the LSTM architecture in terms of both training and validation accuracy scores. A comparative analysis of the proposed model with existing models is presented in Table 4.

**Table 4:** Comparison of the proposed model with existing language identification models.

Author	Models	Features	Performance (Accuracy)
^15^	LSTM and 1D CNN and	no feature identified	95.9%
^16^	FFBPNN	MFCC and PLP	94.6%
^17^	SVM and LDA	MFCC and Formant	93.88% with LDA and 84% with SVM
^19^	CNN	Mel-spectrogram	Naive Bayes achieved 93%, RF achieved 72.42%, SVM achieved 82.88%
^24^	CNN	MFCC, Mel-Spectrogram, Hybrid features	97%, 97.4%, and 99.5% for Testing, Validation and Training accuracy respectively with hybrid feature
^37^	ANN and SVM	Hybrid features	80.82% and 98.06%
^39^	CRNN	Spectrogram	Binary classification achieved 92%, multi-classification achieved 89%
^40^	CNN	spectrogram	92%
^41^	CNN with continuous bag-of-words (CBOW) method	Hybrid features	93.41%
Proposed	LSTM and BLSTM	MFCC	98%

## Conclusion and future work

### Conclusion

This paper presented the development of a language identification (LID) system for Ethio-Semitic languages using Recurrent Neural Networks (RNN). We developed our corpus in order to implement the proposed system because there is no existing large-scale corpus for the four investigated Ethio-Semitic languages for LID purposes. Despite the fact that there are many Ethio-Semitic languages, we chose only four to develop the LID model. The four Ethio-Semitic languages are Amharic, Ge'ez, Guragigna, and Tigrigna. The dataset consists of data obtained directly from native speakers. We used RNN in this work to conduct experiments. We utilized Google Co-laboratory (COLAB) on a CPU with a GPU (Tesla T4, 8 GB) and 12 GB of RAM to do all of the tests. The studies were carried out to examine the results achieved utilizing two extended versions of RNN, LSTM, and BLSTM algorithms, with MFCC Acoustic features for durations of 5 and 10 s. The results achieved show that the most suitable approach for the Ethio-Semitic language dataset was the BLSTM algorithm with the MFCC feature running for 5 s.

### Future work

The study's findings can be used in a variety of fields where spoken language is required. We couldn't readily investigate the suggested system further since there were no resources for other Ethio-Semitic languages. Due to a lack of annotated data for Ethio-Semitic languages, we were forced to limit our investigation to four languages.

For upcoming work, the following are recommended:

- Future studies should try to investigate more Ethio-Semitic languages so as to develop a more robust model for Ethio-Semitic languages.
- This paper used only MFCC as a feature. In future work, we recommend that more features should be used to increase the performance of the model.

## Data Availability

The datasets generated during and/or analyzed during the current study are not publicly available but are available from the corresponding author on reasonable request.

## References

[1] Salau AO, Tamiru NK, Arun D (2022). Image-based number sign recognition for ethiopian sign language using support vector machine”. Lecture Notes in Electric. Eng..

[2] Alemu AA, Fante KA, Info A (2021). Corpus- based word sense disambiguation for Ge’ez language. Ethiop. J. Sci. Sustain. Dev. e-ISSN.

[3] Wondimu M, Tekeba M (2019). “Signal based ethiopian languages identification using Gaussian. J. EEA.

[4] Athiyaa N, Jacob G, Science C, Anna R, College G, Phil M (2019). Spoken language identification system using MFCC features and Gaussian mixture model for tamil and telugu languages. Int. Res. J. Eng. Technol. (IRJET).

[5] Abate ST, Tachbelie MY, Schultz T (2020). Multilingual acoustic and language modeling for Ethio-Semitic languages multilingual acoustic and language modeling for Ethio-Semitic languages. Proc Interspeech.

[6] Belay, B. H. (2021) Deep learning for amharic text-image recognition : algorithm, dataset and application,” 2021.

[7] Feleke TL (2021). Ethiosemitic languages: Classifications and classification determinants. Ampersand.

[8] Hossan, M. A., Memon, S., Gregory, M. A. (2010) A novel approach for MFCC feature extraction. 2010 4th International Conference on Signal Processing and Communication Systems, Gold Coast, 10.1109/ICSPCS.2010.5709752.

[9] Schuster M, Paliwal KK (1997). Bidirectional Recurrent Neural Networks. IEEE Trans. Signal Process..

[10] Aysa, Z. (2022) Language identification-based evaluation of single channel speech separation of overlapped speeches.

[11] Yang, S., Yu, X. (2020) LSTM and GRU neural network performance comparison study,” 10.1109/IWECAI50956.2020.00027.

[12] Gonzalez-dominguez, J., Lopez-moreno, I., Gonzalez-rodriguez, J., Moreno, P. J. Automatic language identification using long short-term memory recurrent neural networks.” 10.1371/journal.pone.0146917 PMC4732772 26824467

[13] Sarraf, A., Azhdari, M., Sarraf, S. A comprehensive review of deep learning architectures for computer vision applications,” pp. 1–29.

[14] Persson, S. (2018) Application of the German traffic sign recognition benchmark on the VGG16 network using transfer learning and bottleneck features in Keras Siri Persson Abstract Application of the German Traffic Sign Recognition Benchmark on the VGG16 network using transf.

[15] Bartz C, Herold T, Yang H, Meinel C (2017). Language identification using deep convolutional recurrent neural networks”. Lect. Notes Comput. Sci..

[16] Deshwal D, Sangwan P, Kumar D (2020). A language identification system using hybrid features and back-propagation neural network. Appl. Acoust..

[17] Anjana JS, Poorna SS (2018). Language Identification from Speech Features Using SVM and LDA. Int. Conf. Wirel. Commun. Signal Process. Networking, WiSPNET.

[18] Hanifa RM, Isa K, Mohamad S (2018). Silence removal from isolated Malay words using framing and windowing method. AIP Conference Proceedings.

[19] Singh G, Sharma S, Kumar V, Kaur M, Baz M, Masud M (2021). Spoken language identification using deep learning. Comput. Intell. Neurosci..

[20] Draghici, A., Abeßer, J., Lukashevich, H. (2020) A study on spoken language identification using deep neural networks. Proceedings of the 15th International Conference on Audio Mostly 10.1145/3411109.3411123

[21] Kong Q, Member S, Cao Y, Iqbal T (2020). PANNs : Large-scale pretrained audio neural networks for audio pattern recognition. IEEE/ACM Trans. Audio, Speech, and Language Process..

[22] Hasan, R., Hasan, M. (2020) Investigation of the effect of MFCC variation on the convolutional neural network-based speech classification. IEEE Region 10 Symposium TENSYMP Dhaka Bangladesh, 10.1109/TENSYMP50017.2020.9230697.

[23] Demilie, W.B., Salau, A.O., Ravulakollu, K.K. (2022) Evaluation of part of speech tagger approaches for the amharic language: a review. 9th International Conference on Computing for Sustainable Global Development (INDIACom), pp. 569–574, 2022. 10.23919/INDIACom54597.2022.9763213

[24] Alemu AA, Melese MD, Salau AO (2023). “Ethio-Semitic language identification using convolutional neural networks with data augmentation. Multimed. Tools Appl..

[25] Maity, S., Kumar Vuppala, A., Rao, K. S., Nandi, D. (2012) IITKGP-MLILSC speech database for language identification. 2012 Natl. Conf. Commun. NCC 2012, 2012. 10.1109/NCC.2012.6176831.

[26] Sandhya, P., Spoorthy, V., Koolagudi, S.G., Sobhana, N.V. (2020) Spectral features for emotional speaker recognition,” third international conference on advances in electronics, computers and communications (ICAECC), pp. 1–3, 2020. 10.1109/icaecc50550.2020.9339502

[27] Aslam MA, Sarwar MU, Hanif MK, Talib R, Khalid U (2018). Acoustic classification using deep learning. Int. J. Adv. Comput. Sci. Appl. (IJACSA).

[28] Abeje BT, Salau AO, Mengistu AD, Tamiru NK (2022). Ethiopian sign language recognition using deep convolutional neural network. Multimed. Tools Appl..

[29] Peche M, Davel MH, Barnard E (2009). Development of a spoken language identification system for South African languages. SAIEE Africa Res. J..

[30] Rao KS, Reddy VR, Maity S (2015). Language identification using spectral and prosodic features. SpringerBriefs in Electric. Comput. Eng..

[31] Mengistu AD (2017). Automatic text independent amharic language speaker recognition in noisy environment using hybrid approaches of LPCC. MFCC and GFCC..

[32] Boucheron, L.E., De Leon, P. L., Sandoval, S. (2011) Hybrid Scalar/ Vector quantization of mel-frequency cepstral coefficients for low bit-rate coding of speech. Data Compression Conference (DCC) - Snowbird, UT, USA, 10.1109/dcc.2011.17

[33] Gupta S, Jaafar J, wan Ahmad WF, Bansal A (2013). Feature extraction using MFCC. Signal & Image Process.: An Int. J..

[34] Pal Singh P (2014). An approach to FCC. IOSR J. Eng..

[35] Chauhan N, Isshiki T, Li D (2019). “Speaker recognition using LPC, MFCC, ZCR features with ANN and SVM classifier for large input database. IEEE 4th Int Conf. Comput. Commun. Syst. ICCCS.

[36] Salau, A. O., Olowoyo, T. D. & Akinola, S. O. Accent Classification of the Three Major Nigerian Indigenous Languages Using 1D CNN LSTM Network Model. *Algorithms for Intelligent Systems*, 1–16. 10.1007/978-981-15-2620-6_1 (Springer Singapore, 2020).

[37] Tamiru NK, Tekeba M, Salau AO (2022). Recognition of Amharic sign language with Amharic alphabet signs using ANN and SVM. Visual Comput..

[38] Demilie, W.B., Salau, A.O. Automated all in one misspelling detection and correction system for Ethiopian languages. J. Cloud Comput. 10.1186/s13677-022-00299-1

[39] Dey S, Sahidullah M, Saha G (2022). An overview of indian spoken language recognition from machine learning perspective. ACM Trans. Asian and Low-Resource Language Inform. Process..

[40] Fesseha A, Xiong S, Emiru ED, Diallo M, Dahou A (2021). Text classification based on convolutional neural networks and word embedding for low-resource languages: Tigrinya. Information.

[41] Furlan B, Batanović V, Nikolić B (2013). Semantic similarity of short texts in languages with a deficient natural language processing support. Decision Support Syst..

